# Oral Microbiome, Oral Health and Systemic Health: A Multidirectional Link

**DOI:** 10.3390/biomedicines10010186

**Published:** 2022-01-17

**Authors:** Elena Maria Varoni, Lia Rimondini

**Affiliations:** 1 Department of Biomedical, Surgical and Dental Sciences, University of Milan, Via Beldiletto 1, 20142 Milan, Italy; 2 Department of Health Sciences, University of Piemonte Orientale, Via Solaroli 17, 28100 Novara, Italy; lia.rimondini@med.uniupo.it

The oral cavity can be regarded as the mirror of systemic health, since many systemic diseases may have manifestations in the oral cavity, as in the case, among oral, potentially malignant disorders, of lupus erythematosus oral lichenoid lesions, and, vice-versa, oral diseases may affect systemic health, impairing patient’s nutrition and wellbeing, reducing the quality of life and increasing stress and anxiety.

Worldwide, oral diseases affect 3.5 billion individuals, and oral and lip cancers rank among the 15 most common oral diseases, with potential repercussions on the systemic health of patients [1]. In this perspective, the World Health Organization recognizes oral health as a “key indicator of overall health, well-being and quality of life” and it shares “modifiable risk factors with the leading noncommunicable diseases (cardiovascular diseases, cancer, chronic respiratory diseases and diabetes)” [1]. An emblematic example is the bi-directional link between diabetes and periodontitis [2]. Strategies to prevent and treat periodontitis should consider a multi-factorial and multi-disciplinary approach, in the perspective of a personalized medicine and of a holistic view; for example, physical exercise, along with nutritional counselling, is a cornerstone in the treatment and prevention of type 2 diabetes, but it can also reduce the prevalence of cardiovascular risk and of periodontal disease [3,4].

One of the most important discoveries of the last decades is the role of the microbiome in the pathogenesis of several systemic and oral diseases, and it may represent a putative component at the base of the bond between oral and systemic health. In 2017, the World Workshop in Oral Medicine designated the “oral microbiome” as a focus area to encourage and promote research related to the development of oral mucosal diseases, including potentially malignant disorders and oral cancer [5,6]. Recently, differences in oral microbial community composition and the functions of patients with oral cancer, with and without lymph node metastases, were also found, showing a potential in patient prognosis [7]. The pre-operative accurate cancer staging of the patient is, indeed, pivotal to identify sentinel lymph node and/or distant metastases, even in the most complicated cases, such as head and neck cutaneous melanoma, which does not always follow standard lymphatic drainage and may benefit from lymphoscintigraphy [8]. 

Nowadays, a growing body of literature supports the finding that oral dysbiosis is implicated in periodontitis, resulting in a personalized pathogenesis, where each individual may experience different microbiome impairments and host risk factors [9,10]. Periodontitis-related oral dysbiosis results in chronic inflammation, which correlates with peculiar inflammatory and vascular patterns, according to the presence of an individual’s risk factors, e.g., smoking habit, gender and age [11], and diabetes [12], as well as specific genetic conditions, as in the case of the hereditary form of gingival fibromatosis [13,14]. In the long term, periodontitis can imbalance pro-inflammatory and anti-inflammatory gene responses, including IL-10 gene polymorphisms and polymorphisms of tumor necrosis factor alpha (TNFα), interleukin 1α-β-RN (IL-1α-β-RN), collagen type-l alpha (COLIA1), and vitamin D receptor (VDRs) genes [15]. 

The oral microbiome has gained more and more interest, also in association with the pathogenesis of several chronic systemic diseases and their treatments. Oral microorganisms can spread to distant body tissues via the blood vessels of the oral cavity or via the gastrointestinal tract, reaching different organs and producing distant local dysbiosis. Recent studies, for instance, have reported a correlation between oral microbiome impairments and an increased risk of pancreatic and liver diseases, with the presence of oral pathogens, predominantly *P. gingivalis*, in the diseased organ [16]. Furthermore, cancer therapies, including chemotherapy, head and neck radiation therapy, or thyroid cancer radioiodine therapy, can damage dental tissues and alter the oral microbiome [17,18,19,20]. In this perspective, innovative strategies for fine tuning the oral microbiome, counteracting dysbiosis, may help in preventing the disease and the complications of therapies. Different approaches may include oral hygiene procedures, prebiotics, probiotics, host response modulators, and the use of nano-sized drug delivery systems to modulate the microbiota [21]. Novel glutathione-stabilized silver nanoparticles (GSH-AgNPs) were demonstrated to possess antibacterial activity against *Porphyromonas gingivalis, Fusobacterium nucleatum*, and *Streptococcus mutans*, with negligible cytotoxic effects when used at low concentrations [22]. Essential oils can also be regarded as promising [23,24,25,26].

In the last decades, literature has also supported the role of the brain–gut–microbiome axis in the etiology of depression and other psychiatric disorders [27], and, similarly, a recent study suggests that the oral microbiome could be implicated in the depression of young adults [28]. Although the impact of the oral microbiota on the psychological health of patients still needs to be better elucidated, oral health is recognized to play a fundamental role in wellbeing and the quality of life [29]. Oral disorders can significantly affect the functional, social and psychological sphere of individuals. Several studies report a reduced quality of life in patients suffering from the most common dental diseases, i.e., periodontitis and caries [30,31], and in patients with oral, potentially malignant disorders or oral cancer [32,33]. Nonetheless, quality of life appears to also be deteriorated in the case of specific systemic conditions, which can affect oral hygiene, such as in hemophilia [34], or in patients experiencing oral complications of particular drugs, including medication-related osteonecrosis of the jaw (MRONJ) [35]. The latter mainly occurs in individuals who receive anti-resorptive drugs (bisphosphonates or denosumab) for the treatment of bone cancer or severe osteoporosis and who need tooth extractions, so a preventive approaches are pivotal [36,37]. The relation between oral disease and psychological or psychiatric disturbances is even closer in the case of burning mouth syndrome (BMS), a neuropathic pain that has psychogenic components [38], for which the management mainly includes the use of psychotropic drugs and psychological support; alternative medicine approaches, based on phytomedicine and acupuncture, are possible, albeit less supported [39,40]. 

The past and future findings about the oral microbiome open new avenues in terms of pathogenesis and treatment, showing an impact on both oral and systemic health, and improving the way we manage patients, contributing towards creating a personalized, multidisciplinary and holistic medicine.

## Data Availability

Not applicable.
